# Hypophosphatemia of prognostic value in acute exacerbation of COPD

**DOI:** 10.1186/cc9789

**Published:** 2011-03-11

**Authors:** N Makhoul, R Farah, L Jacobson

**Affiliations:** 1 Western Galilee Hospital, Naharya, Israel; 2 Ziv Hospital, Zfat, Israel; 3 Naharia Hospital, Naharia, Israel

## Introduction

Phosphorus is the most important anion and it is important to cell function, necessary to create the ATP energy, and an essential component of nucleic acids. Low levels of phosphorus in the blood may be due to a change in functioning of organs participating in the phosphorus balance and affecting the performance of different systems. A low level of phosphorus in the blood increases the exacerbation and the severity of COPD, increasing the need for mechanical ventilation.

## Methods

All patients were hospitalized in our hospital due to acute COPD exacerbation during 6 months. Comparison was made between the group with normal blood phosphorus and the group with a low phosphorus level. We checked the length of hospital stay, the need for ventilation, ventilation duration, mortality and morbidity rates.

## Results

**Figure 1:**
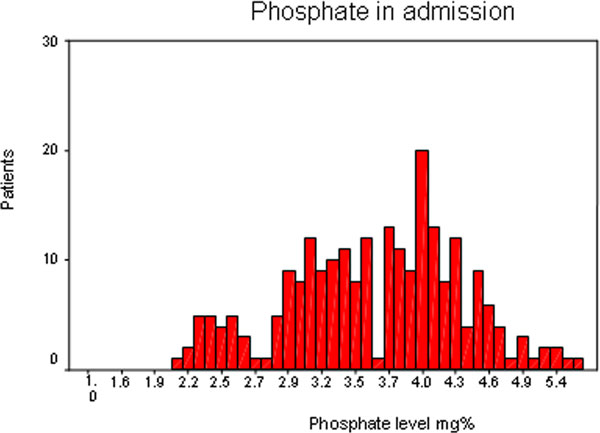
**Phosphate level at admission**.

We examined 242 patients, 73% men 27% women, average age 66.6 years. One hundred and ninety-four patients (80%) were hospitalized in the internal medicine department and 48 (20%) needed mechanical ventilation in the ICU. On admission, 95% of patients had a normal phosphorus level, 5% had a low phosphorus level, in 3.3% the phosphorus level was low, and 1.7% had a very low level of phosphorus. In the group of 48 ventilated patients, in 10% we observed a mild to moderate low phosphorus value and in 8% of patients a very low phosphorus level. See Figure 1.

## Conclusions

Low blood phosphorus levels contribute to increased severity of COPD and the need for ventilation, significantly increase the duration of hospital stay in the ICU, and increase mortality. Correction of these disorders may increase the survival rate of patients with COPD and may improve prognosis.

